# Effects of heat-treatment on the stability and composition of metabolomic extracts from the earthworm *Eisenia fetida*

**DOI:** 10.1007/s11306-016-0967-z

**Published:** 2016-02-05

**Authors:** Tracey B. Schock, Sheri Strickland, Edna J. Steele, Daniel W. Bearden

**Affiliations:** Chemical Sciences Division, Hollings Marine Laboratory, National Institute of Standards and Technology, Charleston, SC 29412 USA; Department of Biology, Chemistry and Physics, Converse College, Spartanburg, SC 29302 USA

**Keywords:** Earthworm, *Eisenia fetida*, Heat stabilization, NMR, Metabolomics, Stability, Tissue extraction

## Abstract

Environmental metabolomics studies employing earthworms as sentinels for soil contamination are numerous, but the instability of the metabolite extracts from these organisms has been minimally addressed. This study evaluated the efficacy of adding a heat-treatment step in two commonly used extraction protocols (Bligh and Dyer and D_2_O phosphate buffer) as a pre-analytical stabilization method. The resulting metabolic profiles of *Eisenia fetida* were assessed using principal component analysis and NMR spectral evaluations. The heated Bligh and Dyer extractions produced stabilized profiles with minimal variation of the extracted metabolomic profiles over time, providing a more suitable method for metabolomic analysis of earthworm extracts.

**Electronic supplementary material:**

The online version of this article (doi:10.1007/s11306-016-0967-z) contains supplementary material, which is available to authorized users.

## Introduction

Terrestrial ecotoxicological studies commonly use earthworms from the *Eisenia* or *Lumbricus* genera for metabolomics analysis. The endogenous metabolites of healthy, non-exposed *Eisenia fetida* have been well characterized (Whitfield Åslund et al. 2011; McKelvie et al. 2011; Yuk et al. 2013; Brown et al. 2008), allowing for potentially straightforward comparison of the metabolic profile of exposed worms. Nevertheless, the level of enzymatic activity in whole-body tissue extracts of earthworms is known to be much higher than that of other species used in ecotoxicological studies (Liebeke and Bundy 2012; Guo et al. 2009). Specifically, earthworm proteases are thought to be particularly problematic when compartmentalized metabolism is disrupted in whole-body analyses. While the analytical reproducibility of nuclear magnetic resonance (NMR) spectroscopy provides consistency in metabolomics measurements (Viant et al. 2009; Ward et al. 2010; Pan and Raftery 2007), preserving the extracted samples from degradation (e.g., enzymatic) during data acquisition, is necessary to prevent alteration of the specific metabolome in question and to reduce the possibility of erroneous findings.

Although there is no consensus on a standardized method of tissue extraction for earthworm metabolomic studies, several extraction protocols have been published (Liebeke and Bundy 2012; Brown et al. 2008; Guo et al. 2009; Lankadurai et al. 2013; Rochfort et al. 2009; Mudiam et al. 2013). Two of the most commonly used extraction procedures are the modified Bligh and Dyer method (Bligh and Dyer 1959; Wu et al. 2008; Liebeke and Bundy 2012) and the D_2_O buffer method (Brown et al. 2008). The Bligh and Dyer method involves bi-phasic extraction of both polar and nonpolar endogenous metabolites using a specific ratio of chloroform, methanol, and water. The D_2_O buffer method involves fewer steps, thus reducing the risk of extraction inconsistencies; however, the polar metabolites extracted include polar lipids.

Working on the hypothesis that the activation of enzymes during tissue extraction might be the cause of NMR sample degradation over time, it may be likely that selective heating alone might be a solution to deactivate these enzymes. The objective of this study was to systematically expand on the work of Liebeke and Bundy, who investigated the impact of extraction protocol parameters, including the impact of filtration and heating of the reconstituted extracts, on the stability of the prepared NMR sample (Liebeke and Bundy 2012). Specifically, the stability was assessed for a 70 % acetonitrile extraction, however, the stability of the four other extraction methods, examined for comprehensive metabolite coverage, was not presented. In this study, we intensively evaluated the two most common extraction techniques, in regard to the stability of the polar extracts for NMR analysis, and determined the impact of various heating protocols during different stages of the extraction process on the stability of the NMR samples.

## Methods

### Animal husbandry and control material preparation

Details of the growth and maintenance of *Eisenia fetida* earthworms and the preparation of a homogenous worm control material (WCM) can be found in Supplemental Information.

### Metabolome extraction and stabilization

#### Extraction

Worm control material (WCM) was extracted with both the modified Bligh and Dyer technique using chloroform/methanol/water (CMW) (Bligh and Dyer 1959; Liebeke and Bundy 2012; Wu et al. 2008) and the D_2_O buffer method (Brown et al. 2008). A general description of each technique is described below, followed by the modifications to optimize stability.

#### CMW

Briefly, 0.025 g of lyophilized WCM was placed into bead beating tubes along with cold methanol (22.2 mL/g dry weight (gdw)) and water (8.9 mL/gdw). Worm wet mass contains approximately 82 % water. The mixture was pulverized using a Precellys 24 homogenizer (Bertin Technologies, France) at 108.3 Hz for two cycles of 15 s with a 15 s rest interval. The homogenate was transferred to a glass vial containing cold chloroform (22.2 mL/gdw) and water (11 mL/gdw), leading to an overall volumetric ratio for the CMW extraction of 2:2:1.8. The mixture was vortexed and then incubated on ice for 10 min. The solvent phases were partitioned by centrifugation at 2000×*g*_n_ at 4 °C for 5 min. The upper polar phase was removed with a pipette and dried by Vacufuge (3 h, at room temperature; Eppendorf, Germany). Samples were reconstituted with 600 µL of 100 mmol L^−1^ D_2_O (phosphate) buffer, pH 7.3, which included 1.0 mmol L^−1^ TMSP (3-trimethylsilyl 2,2,3,3-d_4_ propionate, CAS 24493-21-8) and 0.1 g sodium azide (CAS 26628-22-8) and were immediately analyzed by NMR (550 µL).

#### D_2_O buffer

In 1.5 mL microcentrifuge tubes, 0.025 g WCM was combined with 1.2 mL D_2_O buffer described above. Samples were vortexed for 30 s and sonicated (Branson Ultrasonics, Danbury, CT, USA) in a 25 °C water bath for 15 min. The worm extractions were centrifuged at 14,000×*g*_n_ at 25 °C for 20 min. The supernatant was removed and pipetted into a new tube and centrifuged as before to eliminate residual particulate matter. The supernatant (550 µL) was analyzed by NMR.

### Metabolome stabilization

Samples were extracted in triplicate, subjected to the designated heating protocol (described below) and periodically analyzed by NMR over approximately a 4-day period. Each sample was analyzed by NMR a total of nine times over this period, and the treatment triplicates were staggered in the data acquisition queue to observe time-dependent metabolome changes.

#### CMW

The treatment designations and protocols for the CMW extraction are as follows and are illustrated in Supplemental Information (Fig. S1):

1. N: Control samples which were not treated by heat. These replicates were rehydrated with buffer, then vortexed before 550 μL was placed into the NMR tube.
2. H5: Samples which were heated for 5 min in a 95 °C heat block (Corning, Tewksbury, MA) immediately after rehydration with buffer and subsequently vortexed before 550 μL was placed into the NMR tube.
3. H10: Samples which were heated for 10 min at 95 °C immediately after rehydration with buffer and subsequently vortexed before 550 μL was placed into the NMR tube.
4. VH5: Samples which were vortexed for approximately 30 s immediately after rehydration with buffer and subsequently heated for 5 min at 95 °C before 550 μL was placed into the NMR tube.
5. PH10: Samples which were separated from the organic and tissue layer and heated for 10 min at 60 °C before being dried by Vacufuge. The samples were rehydrated with buffer and vortexed for 30 s before 550 μL was placed into the NMR tube.

#### D_2_O buffer

The treatment designations and protocols for the D_2_O buffer extraction are as follows and are illustrated in Supplemental Information (Fig. S1):

1. N: Control samples which were not treated by heat.
2. DH5: Samples which were heated for 5 min in a 95 °C heat block immediately after buffer addition and prior to sonication.
3. DH10: Samples which were heated for 10 min at 95 °C immediately after buffer addition and prior to sonication.
4. S5: Samples which were heated for 5 min at 95 °C after sonication.
5. C5: Samples which were heated for 5 min at 95 °C, following the final centrifugation and supernatant transfer to a clean microcentrifuge tube.

### ^1^H NMR spectroscopy, spectral analysis and multivariate statistics

All spectra were obtained at 298 K on a Bruker Advance II 700 MHz NMR spectrometer (Bruker Biospin, Inc., Billerica, MA, USA) equipped with a cryoprobe. Complete acquisition and analysis details can be found in Supplemental Information.

## Results and discussion

The objective of this work was to demonstrate the importance of NMR sample stability in order to confidently measure an experimentally-induced metabolomic response. The literature describes two main extraction protocols for metabolite profile analysis of sentinel earthworms in ecotoxicological research of environmental contaminants: the Bligh and Dyer technique using chloroform/methanol/water (CMW) (Bligh and Dyer 1959; Liebeke and Bundy 2012; Wu et al. 2008) and the D_2_O buffer method (Brown et al. 2008). The two techniques differ in that CMW provides complete removal of nonpolar lipids from the polar fraction, whereas the D_2_O buffer method tends to retain more polar lipids, which is visible by the broad NMR spectral resonances and rolling baseline (Fig. S2). The polar macromolecules remaining in the D_2_O buffer extract interact with the other components in the extract and eliminate the resonances of the most abundant compound in worms, HEFS, from the 1D spectra (Bundy et al. 2002) (Fig. S2). The buffer extract also yields higher levels of carbohydrate sugars, which may be evidence of metabolic degradation due to the lengthy extraction protocol at temperatures that are favorable for enzymatic activity and the absence of deproteinizing solvents, like methanol and chloroform, during the extraction (Wu et al. 2008).

### Stability analysis of extracted metabolomes

**Fig. 1:**
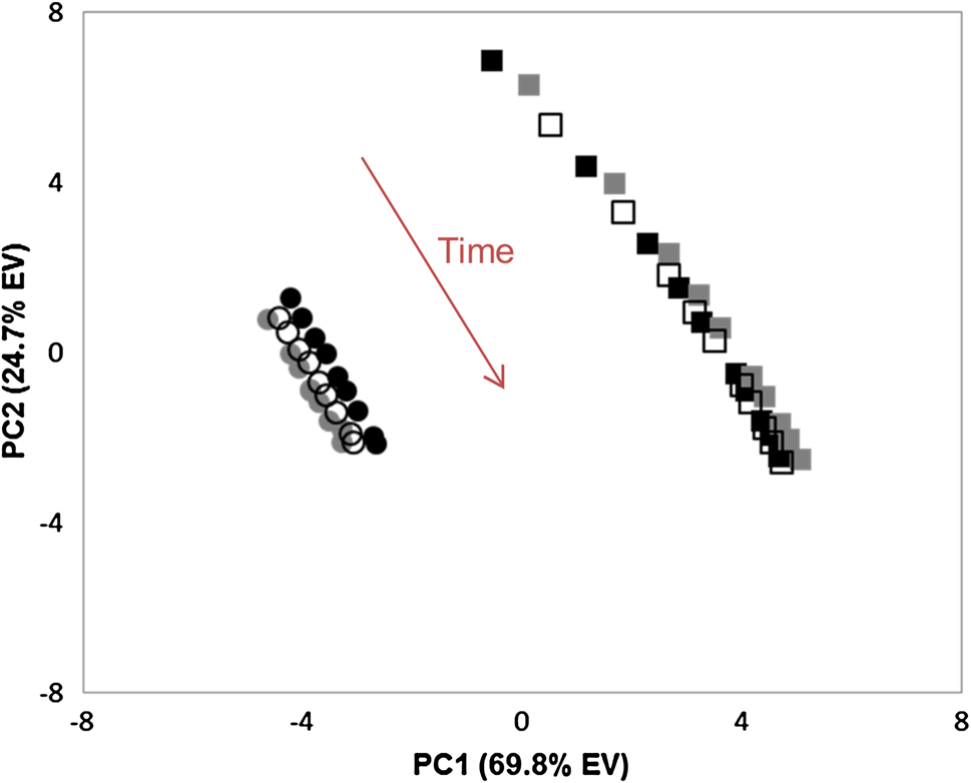
Principal component analysis (PCA) scores plot of worm control material extracted by a chloroform/methanol/water protocol (*circles*) and a D_2_O buffer method (*squares*) in triplicate (1: *black*, 2: *gray*, 3: *open*). Samples were repeatedly analyzed over a 4-day period. The *arrow* shows the direction of metabolic changes with time from unstable samples

Both extraction methods resulted in unstable NMR sample preparations. Analysis of the extracts over time with PCA revealed metabolome and stability differences between extraction methods (Fig. 1). The magnitude of change in the metabolomes of the D_2_O buffer extracted samples was greater than the changes over time for the CMW extracts. Fluctuations were most dramatic during the first six acquired buffer spectra (≈12 h). Visual inspection of the overlaid, repeatedly-acquired spectra supports these observations (Fig. S3). Table S1 lists the most abundant metabolites that changed, either increased or decreased, for both extraction methods. Though some resonances were not clearly visible in the 1D spectra (labeled “not observed” in Table S1), the HSQC verified the presence of these metabolites in the extract. In general, amino acids, organic acids, and carbohydrate levels were most altered. The instability of the D_2_O buffer extracts also showed evidence of biological activity in diminishing resonances of polar lipids over time (Fig. S3).

The challenge with analysis of unstable metabolomes is the potential for misguided biological interpretation of the results. Many of the metabolites listed in Table S1 have been suggested as markers of environmental contaminant effects in several studies (Lankadurai et al. 2013; McKelvie et al. 2011, 2009; Bundy et al. 2008; Brown et al. 2010) and have also been documented to have high relative standard deviations (RSD) within the species, suggesting large inter-individual variation (Whitfield Åslund et al. 2011). In most published reports, experimental methods are often brief and lack essential details, specifically with regard to evidence for extract stability and/or consistency. It is difficult to decipher whether sample stability was of concern during analysis and what measures were taken to minimize these effects; for example, if samples were run in a random order, or if very few were lined up in the queue at one time, or if samples were maintained in the queue at stabilizing temperatures (e.g. 4 °C). Thus, researchers aware of issues regarding sample stability are unable to ascertain whether the large inter-individual variation is a metabolome stability issue or whether the treatment/exposure-caused metabolome changes are justified.

### Metabolome stabilization treatment

**Fig. 2:**
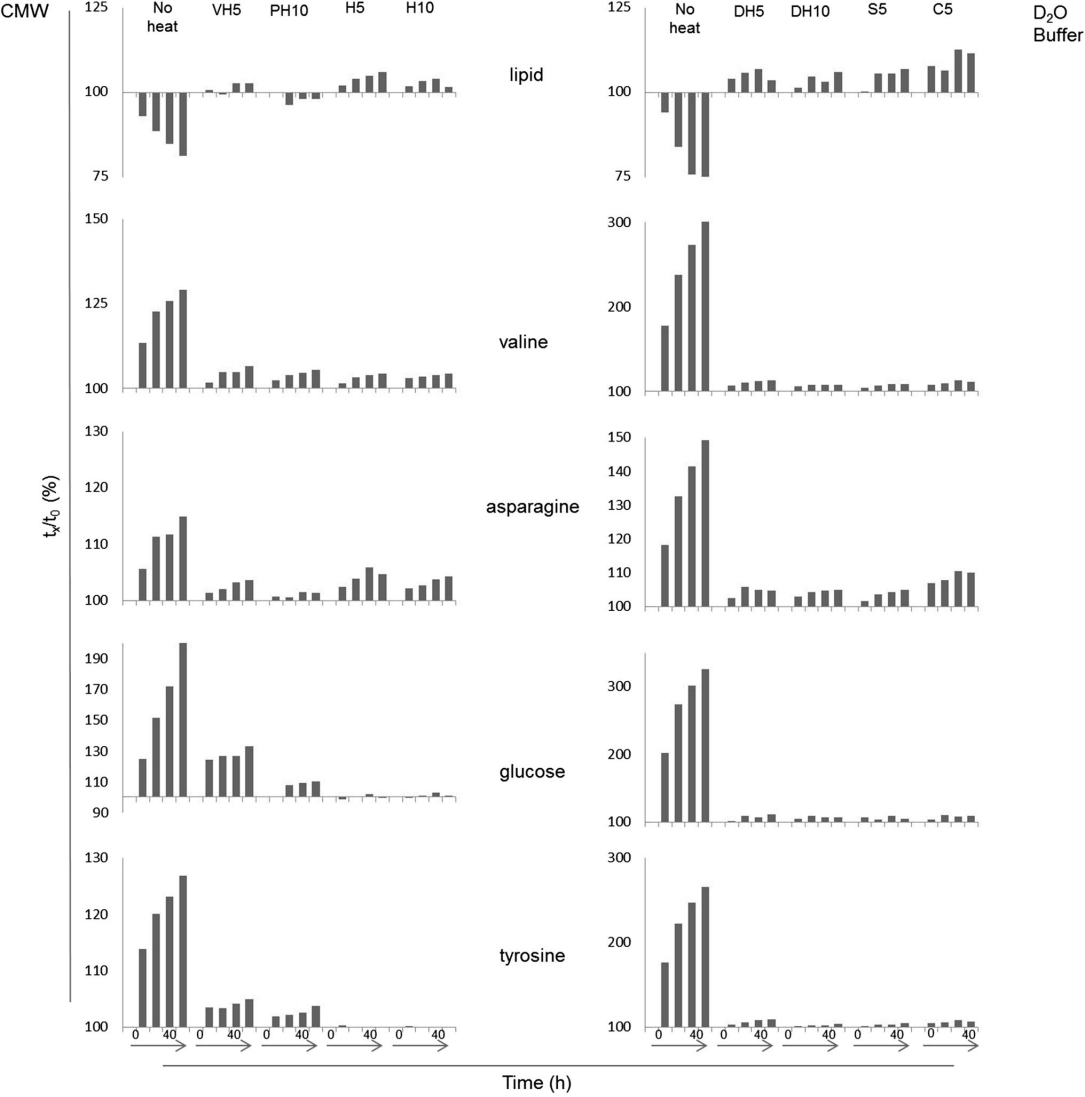
Selected metabolite changes from worm control material extracted by a chloroform/methanol/water protocol (CMW, *left* *panel*) and a D_2_O buffer method (*right panel*). The extracts were treated with heat in order to halt metabolome degradation and were repeatedly analyzed displaying the first 40 h. The change is represented by the percentage of the original measurement

The study which inspired our investigation (Liebeke and Bundy 2012) addressed extraction methods and sample stability for sentinel earthworms, concluding that an acetonitrile/methanol/water extraction provides good metabolite coverage with reproducibility and that a heating and filtering step is required to stabilize the prepared sample. However, the data obtained to confirm the findings of this specific extraction and stabilization method were not included. Stability data was only shown for a 70 % acetonitrile extraction, but not for the four other extraction methods that were comprehensively studied. To shed light on the instability of earthworm extracts and to further examine the effect sample preparation has on metabolite stability, the study detailed here focused on the extraction techniques mentioned above and implemented a heating treatment at various steps to ensure enzymatic denaturation for stabilization of NMR samples. Replication of Liebeke and Bundy’s suggested heating protocol [85 °C for 2 min (Liebeke and Bundy 2012)] failed to eliminate metabolic changes for the CMW extraction (Fig. S4) in our lab. Discussion on the differences in the experiments that may have attributed to differing results can be found in Supplemental Information. Because that treatment did not stabilize the metabolome in this study, we chose to apply a temperature treatment widely used in molecular biology for the denaturation of proteins from their native, active structure (95 °C). The heat treatments were applied at various steps of the extraction and for different lengths of time, including a heat treatment applied to the aqueous fraction of the extracts at a temperature below the boiling point of methanol (60 °C, see Sect. 2 and Fig. S1). A PCA analysis of the two extraction methods, including all heat treatments (Fig. S5a), showed that heating slowed the metabolome changes in both methods in comparison to the dramatic variation observed for the non-heated (N) samples (Fig. 2).

### Effects of heating on D_2_O buffer extracts

The heat treatment applied to the D_2_O buffer extracts just prior to NMR analysis (C5) was most effective in stabilizing the metabolome (Fig. S5b and S6a). This sample was heated when the majority of worm tissue was removed from solution. Heating the buffer extract after sonication and prior to removing worm tissue (S5) was also successful in stabilizing the metabolome (Fig. S5b and S6a). The sonication step likely lysed the intact worm cells releasing active enzymes within the tissue while the subsequent heating step denatured these proteins. The heat treatments (DH5 and DH10) preceding the extraction had more spread in the PCA than C5 and S5, suggesting small continuing metabolome changes with time (Fig. S5b and S6a).

Visual inspection of the heat-treated, D_2_O buffer extracted spectra showed slight spectral changes in all treatments compared to the large changes of the non-heated samples. Interestingly, the NMR internal standard peaks (TMSP) of the C5 group had peak areas approximately 60 % of the peak areas of the non-heated and the other heat-treated samples (Fig. S7). Results of these observations are discussed in Supplemental Information and the data suggests eliminating C5 as a feasible protocol.

The heat-treated D_2_O buffer extracts were more reproducible and stabile than the non-heated extracts (N; Fig. S5b, 2 and S6a); however, the original metabolic profile was altered by these treatments. A loadings plot corresponding to a PCA of the initial non-heated buffer extracts and all the heat-treated buffer extracts (Fig. S6b) showed decreases in sugars and amino acids and an increase in lipids as the heat treatments shifted the metabolome from the initial non-heated profile (Fig. S8). The C5 data in this analysis displays the error in the potential quantitation of metabolite levels as a result of worm component interaction with the NMR internal standard.

### Effects of heating on CMW extracts

The CMW heat treatment profiles did not show much variation in the extraction profiles and were more closely matched with the profiles of the corresponding N samples (Fig. 2, S5b, S8 and S9a), providing a stronger case for this extraction method. Much of the variability between CMW samples in PC1 corresponded to the resonances of the compound HEFS. The time evolution of the spectra is mostly contained in PC2, except for the group heated prior to evaporation of extraction solvents (PH10), whose random sequence suggested lack of time dependence until PC3 (not shown). This analysis was repeated after excluding the spectral peaks from HEFS. Again, the non-heated samples changed dramatically with time (data not shown), but now the treated samples covered a reduced PC area (Fig. S9b). Removing HEFS from the analysis reversed the time sequence for PH10 and VH5 samples and shifted the time changes of the rehydrated and heated samples (H5 and H10) from the PC2 to the PC1 axis, demonstrating the possible complications and interference of this compound in metabolomics type analyses. For further discussion on HEFS in worm extracts, see Supplemental Information.

To determine the optimal treatment for preservation and stabilization of the intended metabolic profile, the CMW extracts were compared pairwise (excluding HEFS) and the spectra were visually examined. In many of the pairwise comparisons, where a PCA separation was visible (data not shown), the models were not terribly strong; and the loadings plots were uninformative indicating that small, random differences were causing the variation in the spectra. Overall, time dependent spectral changes in the heat treatments of CMW samples were slight (Fig. 2) and were reflected by variations of very small peaks, such as the increasing unidentified peak at 1.15 ppm (Fig. S11). Compared to the corresponding N samples, in general, most metabolites were reduced by heating (Fig. S8).

Our observations suggest the H5 and VH5 treatments are generally equivalent, with the exception that VH5 displays small time-dependent glucose changes (Fig. 2) and thus H5 would be the suggested method. There was not a compelling reason to extend the heating time (H10).

## Conclusions

This study aimed to identify an extraction technique for a sentinel toxicological species (*E. fetida*) that offers broad coverage of the aqueous metabolome with an appropriate heat treatment to stabilize the metabolome during data acquisition. All heat treatments reduced the time-dependent change in the spectra significantly compared to the non-heated samples. The heat treatments of the D_2_O buffer extracts altered the reference metabolome more drastically than the treatments applied to CMW extracts. The D_2_O buffer extracts also showed evidence of worm components interacting with the NMR internal standard and HEFS, which compromises the ability to quantify metabolomic effects of a treatment, and consequently we suggest quantitation with D_2_O buffer extracts be avoided. Our results show that the procedure to produce the most unadulterated, stable profile is a CMW extraction with a short heat treatment (H5). Stability analyses should be considered for all metabolomics studies using tissue extracts or biofluid samples.

## Electronic supplementary material

Supplementary material 1 (DOC 863 kb)
