# Supplementary material for: Effects of heat-treatment on the stability and composition of metabolomic extracts from the earthworm Eisenia fetida
Source: Metabolomics. 2016 Feb 5;12:47. doi: 10.1007/s11306-016-0967-z (PMC4744258; doi:10.1007/s11306-016-0967-z)
Supplement: Supplementary file 1 — Supplementary material 1 (DOC 863 kb) [file 11306_2016_967_MOESM1_ESM.doc]

**Supplemental Information**

**Effects of Heat-treatment on the Stability and Composition of Metabolomic Extracts from the Earthworm *Eisenia fetida***

Tracey B. Schock^1^, Sheri Strickland^2^, Edna J. Steele^2^, and Daniel W. Bearden^1^

^1^ Chemical Sciences Division, National Institute of Standards and Technology, Hollings Marine Laboratory, Charleston, SC 29412

^2^ Department of Biology, Chemistry and Physics, Converse College, Spartanburg, SC 29302

**Methods**

*Animal Husbandry*

*Eisenia fetida* earthworms were used because they are the test species recommended for toxicity testing by the Organization for Economic Cooperation and Development (OECD, 1984). The earthworms were purchased from The Worm Factory (Kingston, ON, Canada). Worms were raised in two 10-gallon glass aquaria containing Magic Worm® bedding (sphagnum peat; Magic Products, Amherst Junction, WI, USA). The bedding was first prepared, according to manufacturer’s instructions, by mixing 1 L of deionized water with every 500 g of dry bedding; the worms were added 24 h later. Initially, 300 worms were added to 1 kg of dry bedding per aquarium. The bedding was sprayed liberally with deionized water at every feeding to maintain its moisture content, and it was completely replaced every three months to ensure a healthy environment for the worms. The earthworms were maintained at 20 °C to 22 °C; the optimal temperature for *E. fetida* is 20 °C to 29 °C (Presley et al. 1996). The worms were fed three times per week on a diet of Magic Worm food, which contained approximately 12.0 % crude protein, 1.0 % crude fat, and 6.0 % crude fiber.

*Control Material Preparation*

A homogenous worm control material (WCM) was prepared to investigate the stability of various extracted metabolomes. Forty worms ranging in size from 0.300 g to 0.400 g were gently rinsed with deionized water and then depurated for 96 h in 473.18 cm^3^ glass jars with the bottom lined with Whatman #1 filter paper. The worms were then flash frozen in liquid nitrogen and stored at -80 °C. The worms were lyophilized and homogenized in batches of six individual worms using a chemically cleaned mortar and pestle. All batches were ultimately combined and the powder was mixed thoroughly by vortex in a 50 mL centrifuge tube and stored at -80 °C.

*^1^H NMR Spectroscopy*

All spectra were obtained at 298 K on a Bruker Advance II 700 MHz NMR spectrometer (Bruker Biospin, Inc., Billerica, MA) equipped with a cryoprobe (TCI 5 mm triple-resonance, z-gradient). Spectra were collected under full automation using ICON-NMR with a standard 1D pulse sequence (noesygppr1d) containing a spoiler gradient and water suppression. ^1^H Spectra were acquired into 65536 real data points across a spectral width of 20 ppm with 8 steady state scans, 80 transients, a 3 s relaxation delay, and a 60 ms mixing period. The spectra were Fourier transformed after multiplying the free induction decay by an exponential line broadening function of 0.3 Hz and zero-filling to 65536 complex points. The spectra were manually phased, and the baseline was automatically corrected.

Two-dimensional edited heteronuclear single quantum correlation (HSQC) spectra with adiabatic ^13^C decoupling were collected to enhance metabolite identification. In general, 128 scans and 2048 data points with 512 increments were acquired with spectral widths of 11 ppm in F2 and 180 ppm in F1 (^13^C). A relaxation delay equal to 1.5 s was used between acquisitions, and a refocusing delay of 3.45 ms was implemented. The FIDs were weighted using a shifted sine bell function in both dimensions. Manual two-dimensional phasing was applied; all spectra were referenced to the TMSP internal standard at 0.00 ppm for ^1^H and ^13^C.

*NMR Spectral Analysis and Multivariate Statistics*

Earthworm metabolites were identified based on 1D ^1^H and 2D ^1^H-^13^C NMR experiments. Identities were based on comparison of chemical shifts and spin-spin couplings with reference spectra and tables noted in published reports(Bundy et al. 2002; Yuk et al. 2013), the Human Metabolome Database (HMDB)(Wishart et al. 2007), the Biological Magnetic Resonance data bank (BMRB)(Ulrich et al. 2008), an in-house compiled database, the databases used with AMIX 3.9.14 (Bruker Biospin), and Chenomx® NMR Suite profiling software (version 7.7).

For stability analysis, the ^1^H NMR earthworm spectra were evaluated by binning the spectra at 0.005 ppm bins from 0.2 ppm to 10 ppm excluding water (4.7 ppm to 5.0 ppm), chloroform (7.67 ppm to 7.69 ppm), and resonances of 2-hexyl-5-ethyl-furansulfonate (HEFS)(Bundy et al. 2002) for specific analyses described below using AMIX. Principal components analysis (PCA) was conducted using spectra normalized to constant total spectral area of the included regions with mean-centered, pareto-scaled bins to show trends and stability of extracted earthworm metabolomics data. Spectral changes within and among treatment groups were assessed by direct comparison of overlaid spectra. Selected metabolites, with single compound resonances, were chosen to calculate heat treatment effects using the integration tool in AMIX.

**Results and Discussion**

*Heat Treatment of 85 °C*

Replication of Liebeke and Bundy’s suggested heating protocol (85°C for 2 min (Liebeke and Bundy 2012)) failed to eliminate metabolic changes for the CMW extraction (Fig. S4) in our lab. In addition to the differences in extraction solvents used, the studies also differed in the genus of worm (*Lumbricus rubellus*) and state of the starting tissue material. Where Liebeke and Bundy (Liebeke and Bundy 2012) used a wet, homogenized worm tissue, this experiment used a dried, homogenized tissue. Both methods utilized a mortar and pestle technique for tissue disruption and, practically, a wet tissue can be more thoroughly homogenized than a dry tissue. The tough cuticle of dried worms is difficult to grind to a fine, consistent powder by this method. Thus, the incomplete tissue disruption resulted in a smaller tissue surface area to be in contact with the extraction solvents. Bead beating was applied in an effort to increase the surface exchange required for optimal extraction, but this did not aid in stabilizing the extracted metabolome which still changed after heating at 85 °C. Moreover, we have observed sample instability in other organism studies when dried, homogenized, whole-body analyses were conducted. It is our recommendation that wet tissue (when logistically possible) be used for homogenization and analysis, in order to produce an unaltered, standardized material that best represents the original sample.

An earthworm is a poikilotherm whose metabolism is affected by external temperatures, and the native environmental temperature of worms ranges from 12 °C to 30 °C (Khan et al. 2012). Enzymes catalyze biochemical reactions at physiological temperatures of an organism; therefore, Liebeke and Bundy (Liebeke and Bundy 2012) hypothesizing that 85 °C would denature all *in vivo* earthworm enzymes is a warranted suggestion. However, because that treatment did not stabilize the metabolome in this study (Fig. S4), we chose to apply a temperature treatment widely used in molecular biology for the denaturation of proteins from their native, active structure (95 °C).

*Extract Interaction with NMR Internal Standard*

The NMR internal standard peaks (TMSP) of the C5 group had peak areas approximately 60 % of the peak areas of the non-heated and the other heat-treated samples (Fig. S7). The C5 TMSP peaks also had broader line widths (ν_1/2_) with a mean of 3.26 Hz ± 0.10 Hz compared to the mean of 1.48 Hz ± 0.09 Hz for the remaining samples (p<0.001, *t*-test, α= 0.05). It is well known that proteins interact with TMSP (also DSS, an alternate NMR internal standard) and thus its use in quantitative metabolomic analysis of unprocessed biofluids is not advised (Bell et al. 1989; Kriat et al. 1992; Nowick et al. 2003). Tissue extracts, like the D_2_O buffer method described here, should logically fall into this same category; the extraction comprises solvent containing TMSP and highly proteinaceous worm tissue. Compared to the TMSP peaks of the CMW extracts (mean ν_1/2_= 1.27 Hz ± 0.09 Hz), which are deproteinized prior to the addition of the buffer containing TMSP, the D_2_O TMSP peaks, again, have significant line broadening (p<0.001, *t*-test, α= 0.05). It is important to note that both extraction methods were prepared using the same buffer preparation with a ν_1/2_ of 1.18 Hz. The question remains, however, as to what caused the significant interaction with TMSP in the C5 extracts. Of all the buffer extracts, these samples were the only group void of worm tissue prior to the heat treatment. Additionally, it was observed that these samples also became cloudy and turbid after heating.

Pegos and colleagues came across a similar situation in experiments with the bacteria *Xanthomonas axonopodis* pv. *citri 306,* which produces a polysaccharide matrix known as xanthan gum (Pegos et al. 2014). They observed possible xanthan gum reactivity with the NMR standard. Likewise, earthworms excrete copious amounts of mucus composed of water, electrolytes, glycoproteins, mucopolysaccharides and larger compounds such lectins(Jamieson 1981). We hypothesize that heating the sample may have enhanced interactions with solubilized mucus components and TMSP, thus eliminating C5 as a feasible protocol.

*Extraction of HEFS from D_2_O Buffer Extracts*

Though HEFS played a large part in sample variability, possibly due to tissue inhomogeneity, Liebeke and Bundy state that it does not have an impact on stability (Liebeke and Bundy 2012). Thus, this work did not consider the removal of HEFS from samples. The Liebeke and Bundy cleanup for HEFS is a time consuming step carrying the possibility for loss of valuable metabolite information. We did, however, re-extract one NMR sample from each D_2_O buffer extraction group as per the CMW protocol to determine the presence of HEFS. To the NMR sample (550 µL), 611 µL of each chilled methanol and chloroform were added, vortexed, incubated on ice, and the phases were separated. The aqueous layer was dried and rehydrated in D_2_O. The buffering components, as well as the TMSP, remained in the sample from the previous preparation. The re-extraction removed macromolecules such as proteins and lipids from the D_2_O buffer samples revealing HEFS resonances (Fig. S10).

**Tables and Figures**


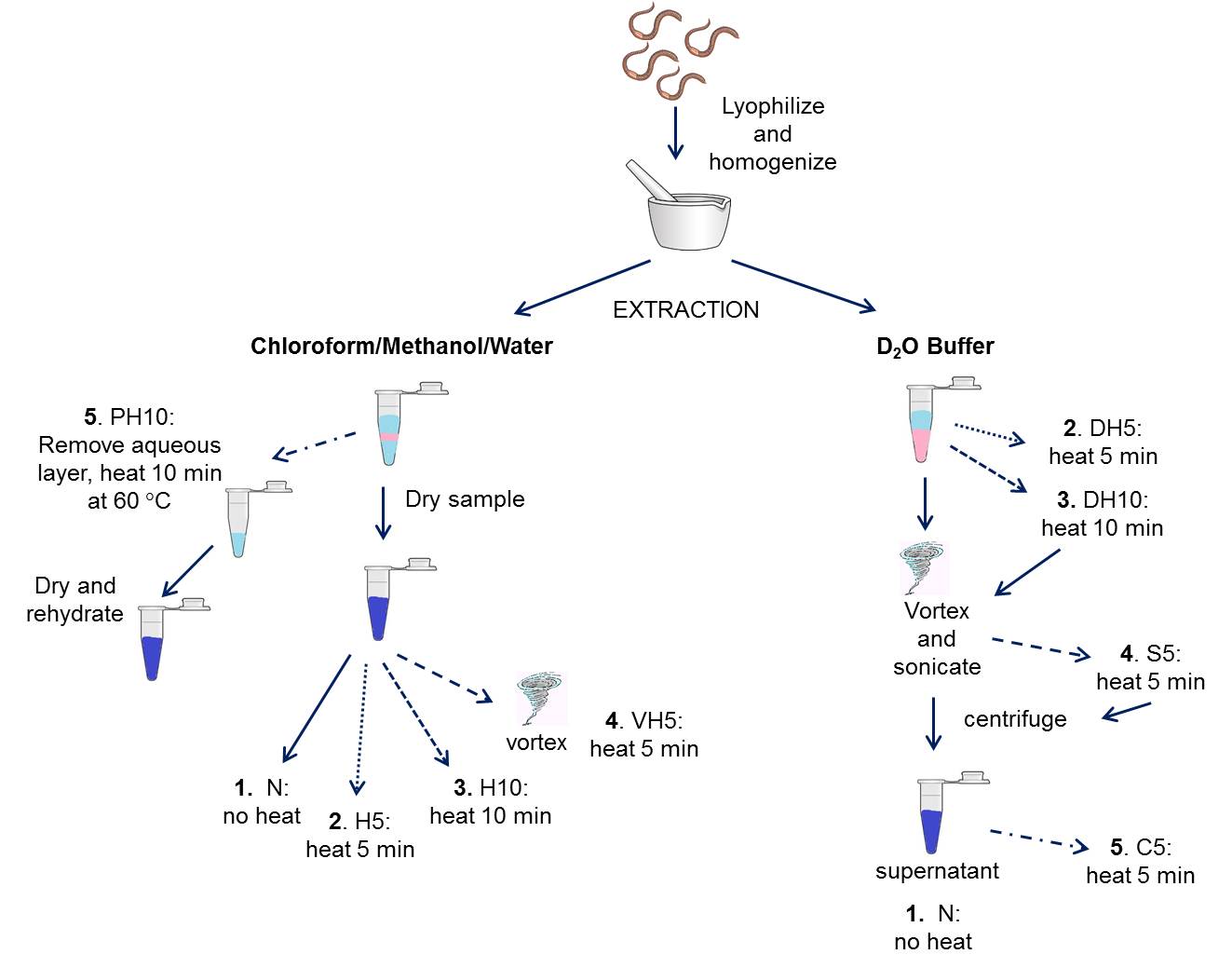


Figure S1. Schemata of stability study for extraction of worm control material by chloroform/methanol/water (CMW) or D_2_O buffer. **1** corresponds to the typical non-heated extract analyzed by NMR. Steps applying heat-treatments are distinguished **2-5**.

**
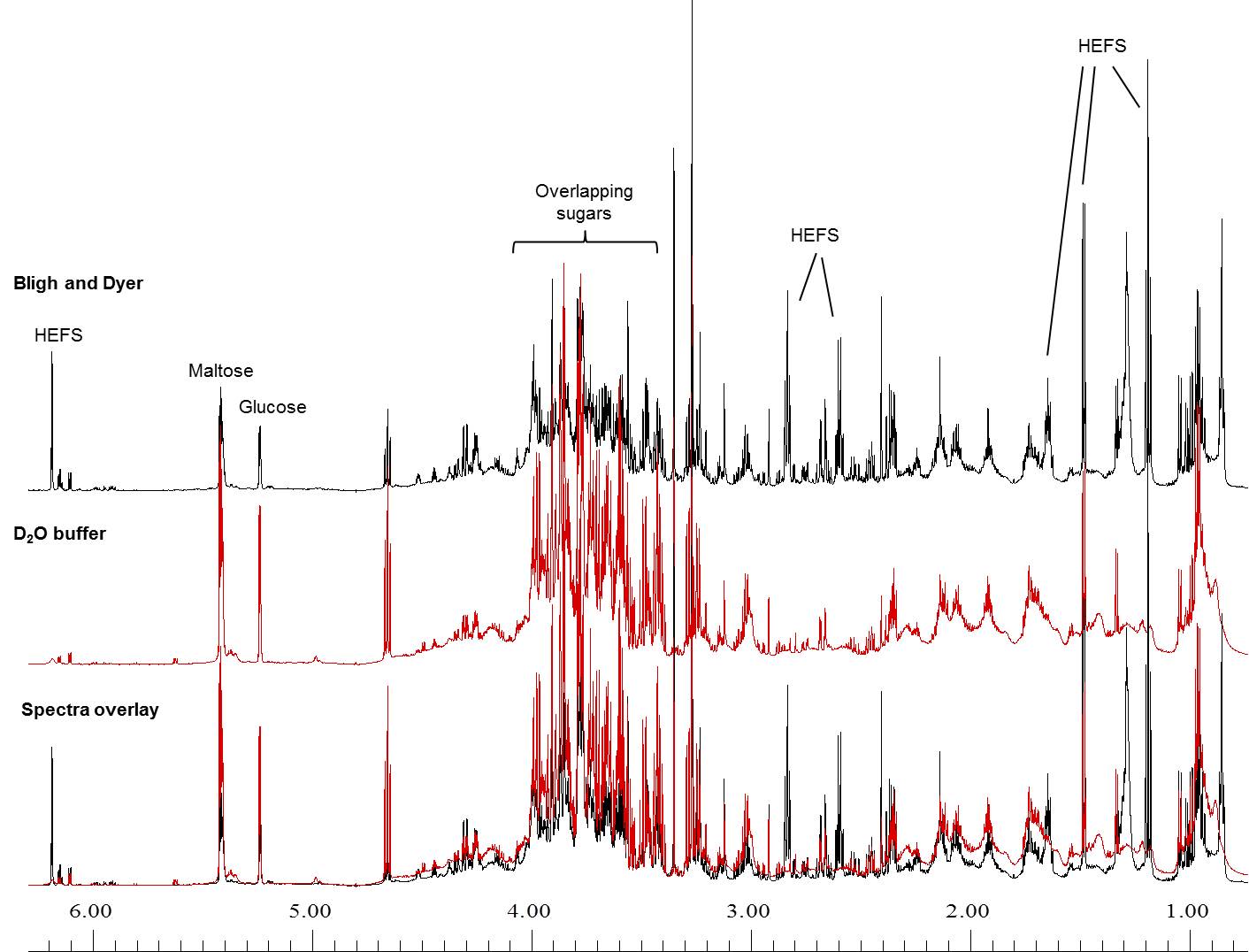
**

ppm

Figure S2. ^1^H NMR spectra of a homogeneous worm control material extracted by a modified Bligh and Dyer chloroform/methanol/water protocol (top, black) and a D_2_O buffer method (middle, red). A direct comparison of metabolite profiles from each extraction method is shown by overlaying spectra (bottom) and metabolite differences are annotated.


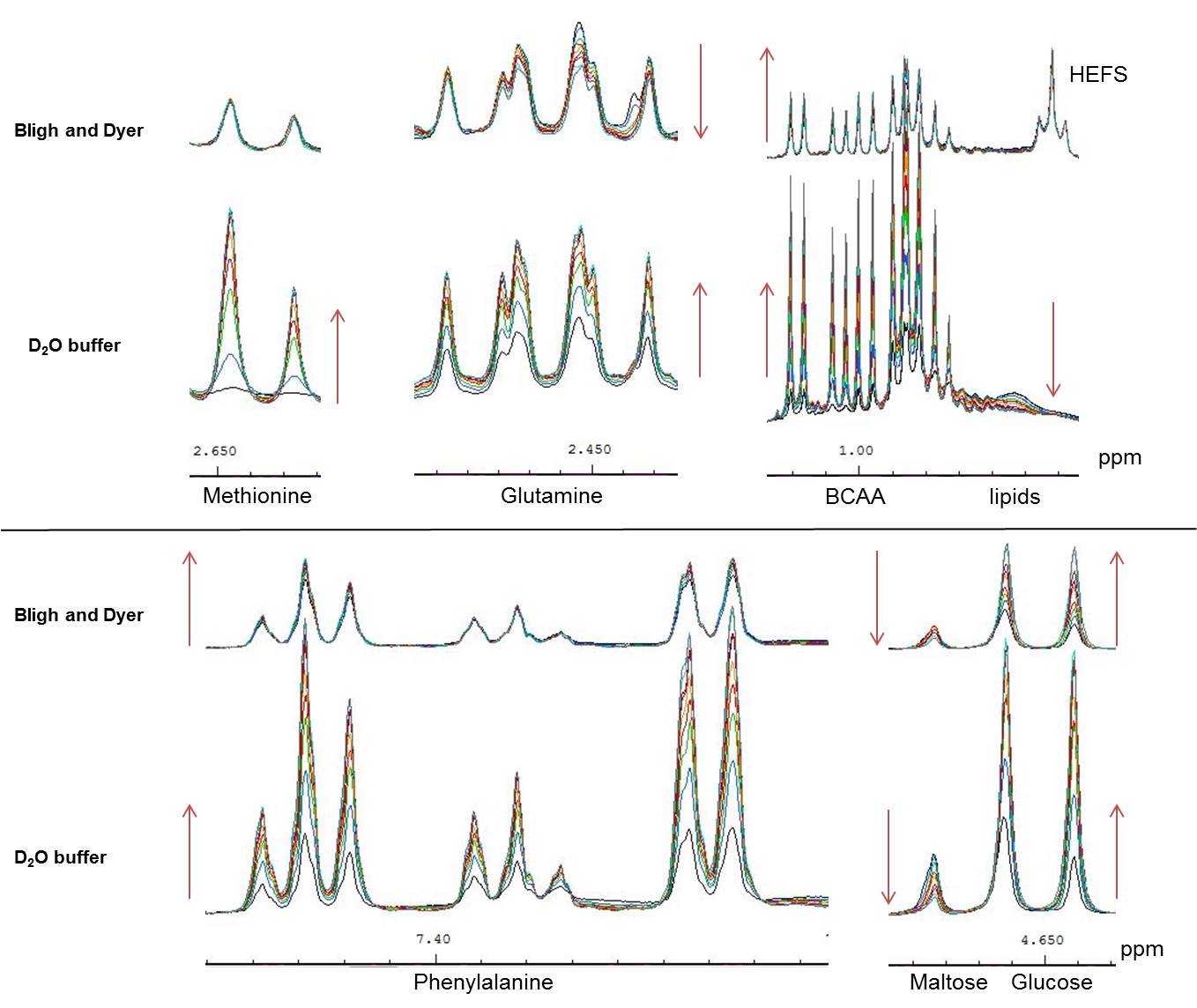


Figure S3. Overlaid snapshots from five regions of the ^1^H NMR spectra of worm control material extracted by CMW (top spectra) or D_2_O buffer (bottom spectra) and analyzed repeatedly over a four day period. The time sequence appears as black>blue>green>red>orange>purple>maroon>aqua>gray. Arrows denote metabolites increasing or decreasing with time. The absence of an arrow suggests no metabolite changes.

Table S1. Altered metabolites due to sample instability from ^1^H NMR spectra from two extraction methods, chloroform/methanol/water (CMW) or D_2_O buffer. Metabolites increased with time (+), decreased with time (-), or did not change with time. Several metabolites were observable in only one extraction method (n/o: not observed). Metabolite identification was verified from spectra referenced to TMSP with Chenomx software and 2D ^1^H-^13^C HSQC.

^a^ Metabolite regions with visible spectral instability, multiplicity d: doublet, dd: doublet of doublets, m: multiplet, s: singlet, t: triplet.


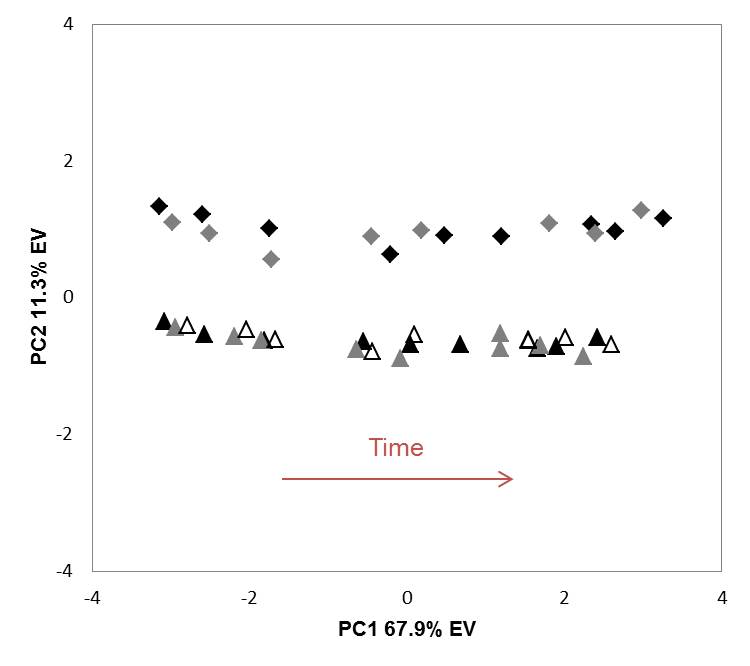


Figure S4. Principal component analysis (PCA) scores plot of worm control material extracted by a chloroform/methanol/water protocol then heated at 85°C (triangles) or not heated (diamonds) (1: black, 2: gray, 3: open; one sample lost) and were repeatedly analyzed over 55 hours. The arrow shows the direction of metabolic changes with time from unstable samples.

**
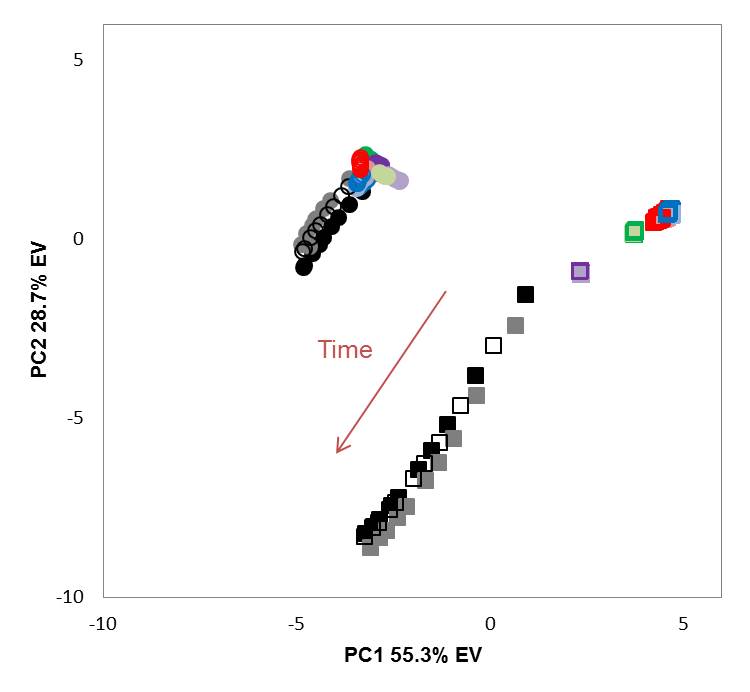
**

A


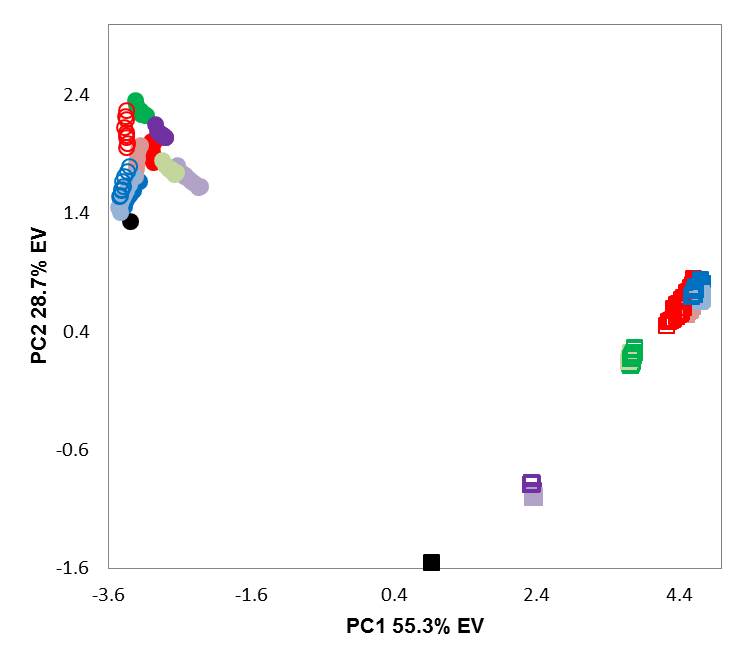


B

Figure S5. (A) Principal component analysis (PCA) scores plot of worm control material extracted by a chloroform/methanol/water protocol (circles, N) and a D_2_O buffer method (squares, N) in triplicate (1: dark, 2: light, 3: open), treated with heat in order to halt metabolome degradation (circles: VH5-red, PH10-blue, H5-green, H10-purple; squares: DH5-red, DH10-blue, S5-green, C5-purple) and repeatedly analyzed over a four day period. The arrow shows the direction of metabolic changes with time in unstable samples. (B) Expansion of the PCA above showing the heat treated samples and the first N samples for each extraction method.


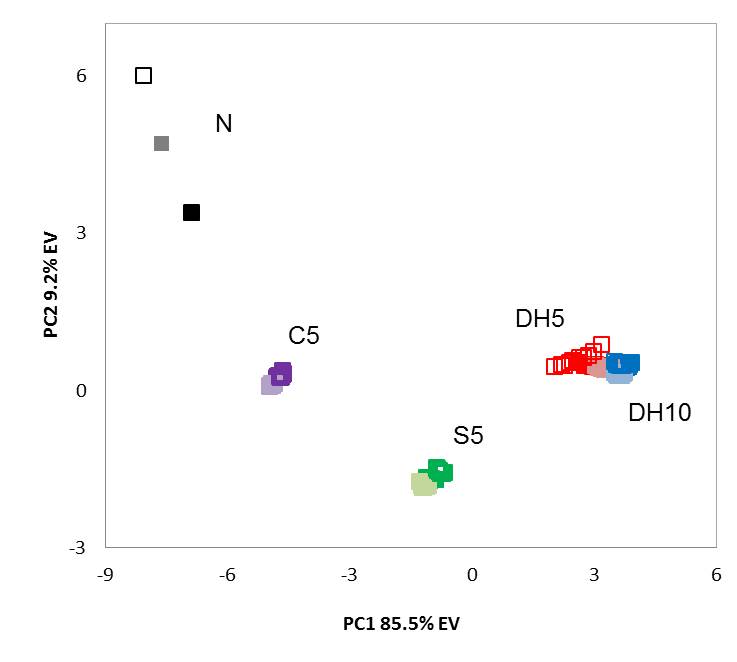


A


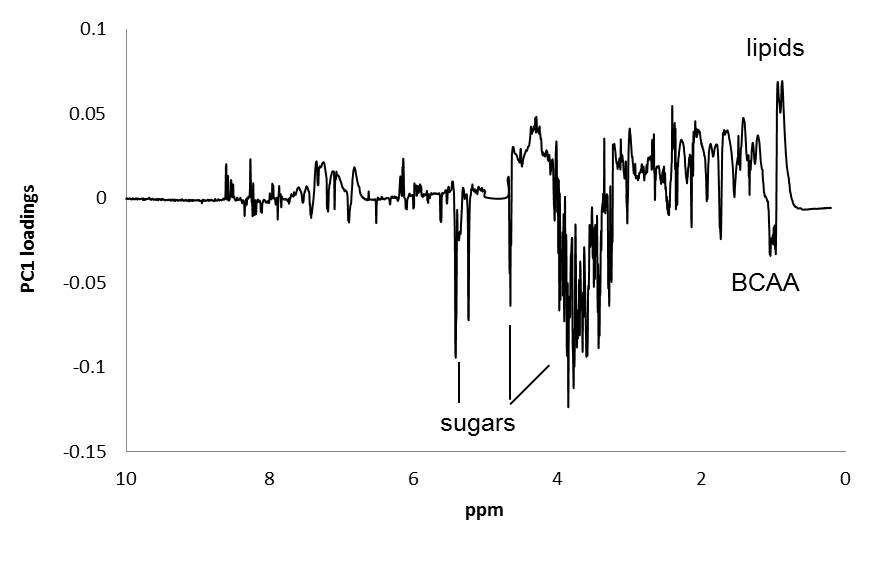


B

Figure S6. (a) Principal component analysis (PCA) scores plot of worm control material extracted by D_2_O buffer method (squares, N) in triplicate (1: dark, 2: light, 3: open), treated with heat in order to halt metabolome degradation (squares: DH5-red, DH10-blue, S5-green, C5-purple) and repeatedly analyzed over a four day period. (b) PC1 loadings plot showing metabolite changes due to heat treatments. BCAA: branched chain amino acids.


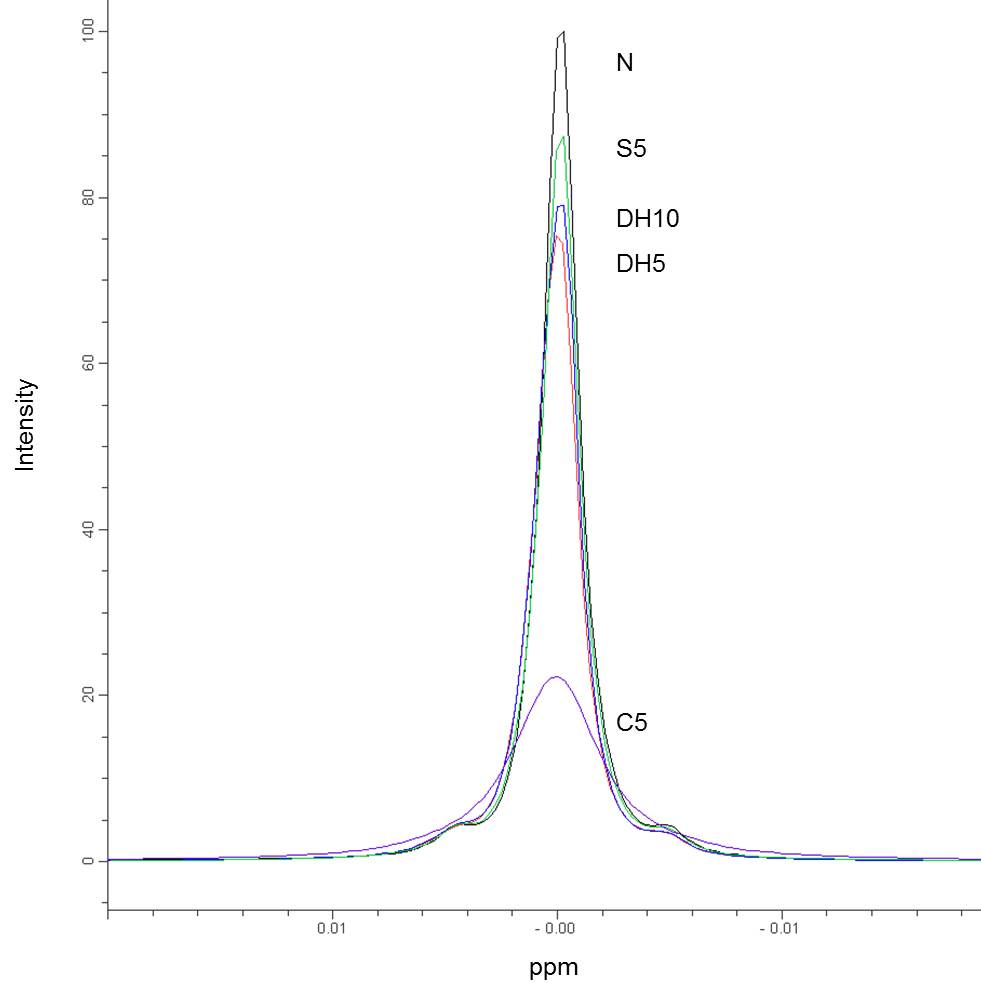


Figure S7. NMR chemical shift standard TMSP (0.0 ppm) from representative ^1^H NMR spectra of worm control material extracted by D_2_O buffer (N, black) and treated with various heating protocols (DH5-red, DH10-blue, S5-green, C5-purple).


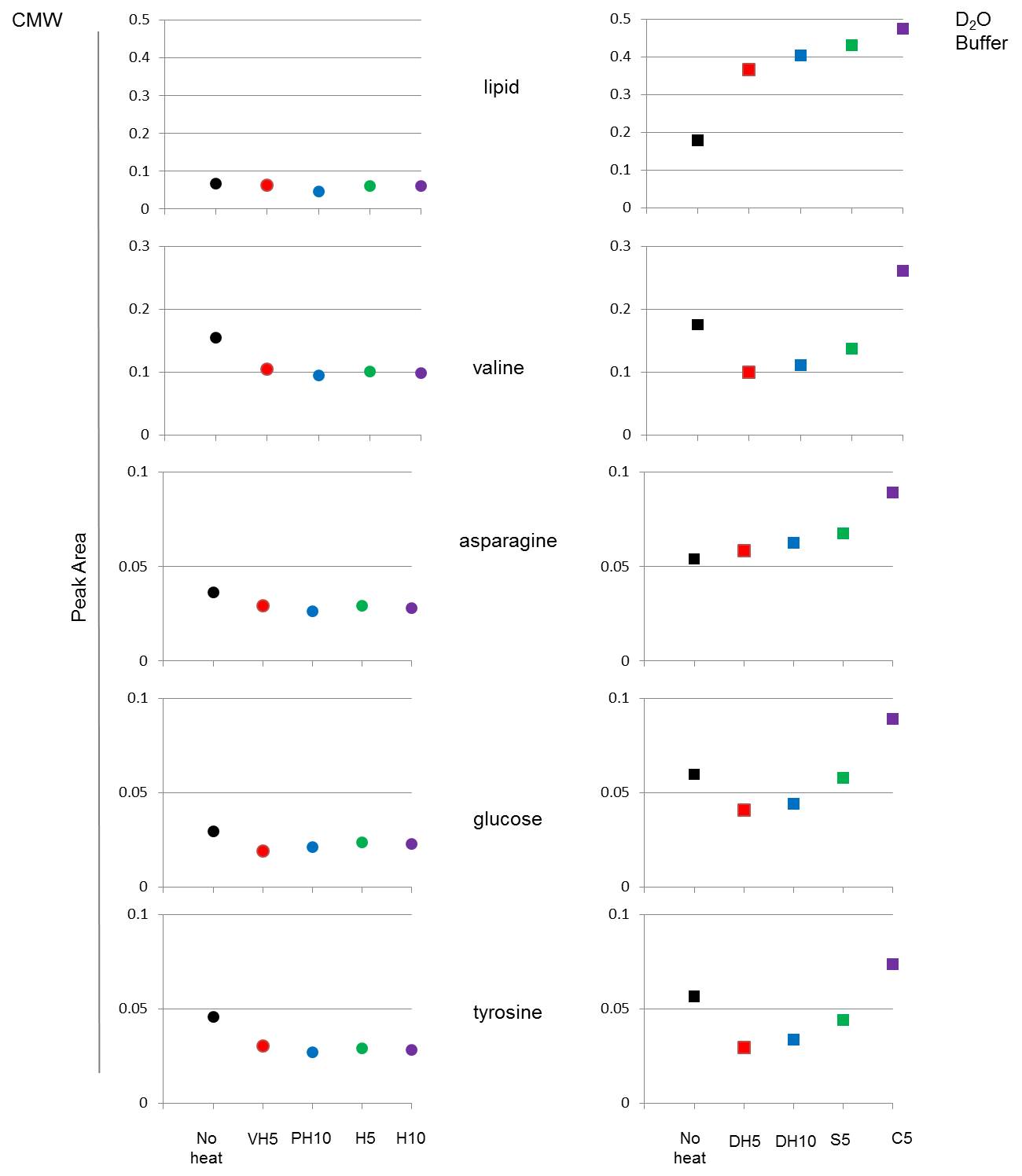


Figure S8. Response of the worm metabolome to heat treatment. Normalized (to TMSP) NMR peak areas for selected metabolites of worm control material extracts (CMW or D_2_O buffer) treated with and without heat. Symbols represent the first NMR experiment run for each treatment. The graphs reflect how heat treatments alter the metabolomics profile in comparison to the initial non-heated samples (n = 1).


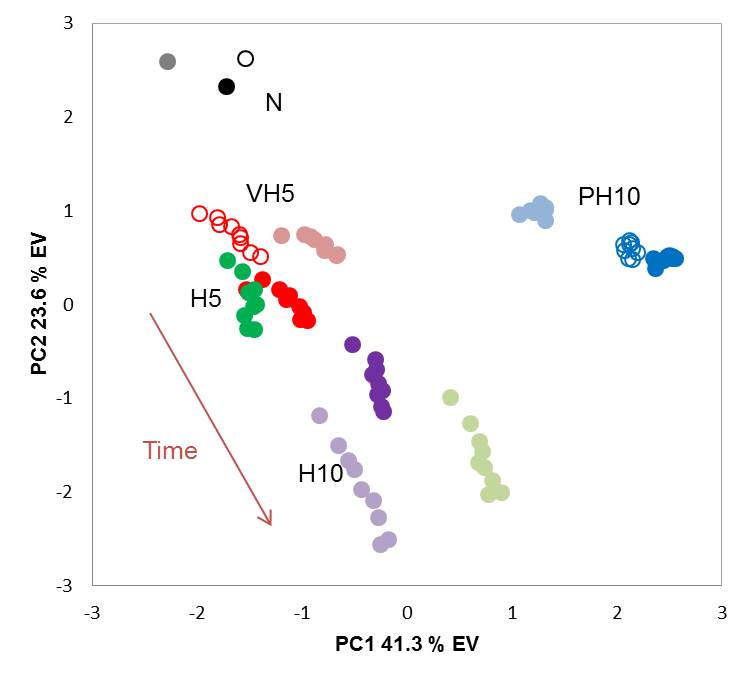


A


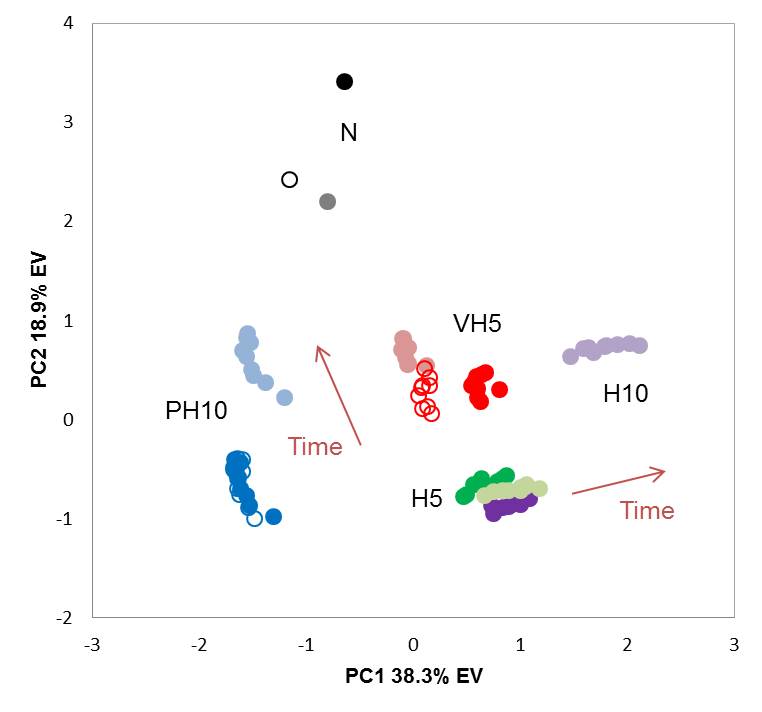


B

Figure S9. (a) Principal component analysis (PCA) scores plot of worm control material extracted by a chloroform/methanol/water protocol (circles, N) in triplicate (1: dark, 2: light, 3: open), treated with heat in order to halt metabolome degradation (circles: VH5-red, PH10-blue, H5-green, H10-purple) and repeatedly analyzed over a four day period. (b) PCA analysis of the same material excluding the compound HEFS from analysis. Arrows denote direction of metabolic change with time.


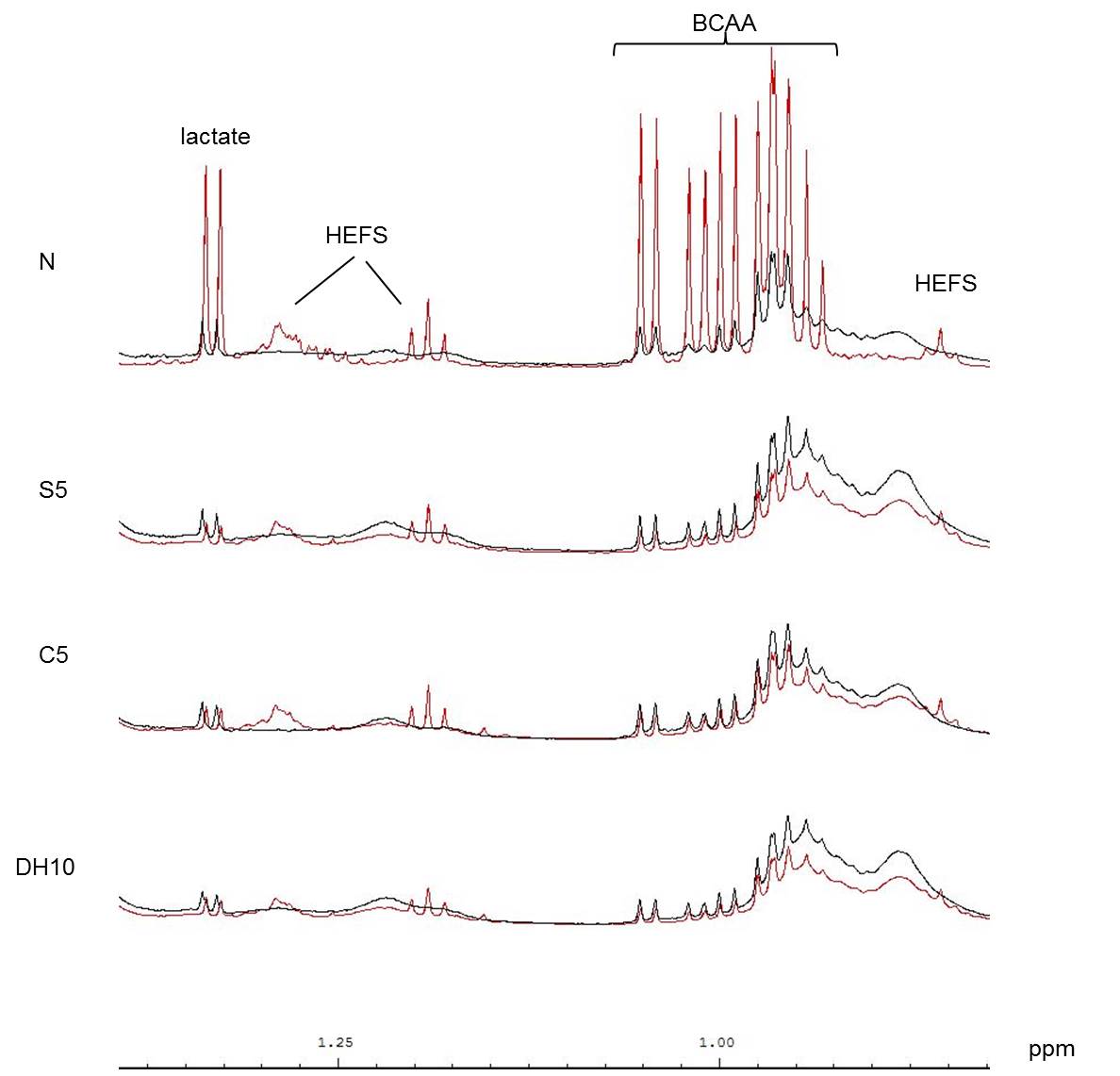


Figure S10. A portion of the aliphatic region of ^1^H NMR spectra from worm control material extracted by D_2_O buffer (black) and treated with various heating protocols. The NMR samples were then re-extracted using a chloroform/methanol/water protocol (red) showing the presence of HEFS. Spectra were normalized to the TMSP peak (0.0 ppm). BCAA: branched chain amino acids.


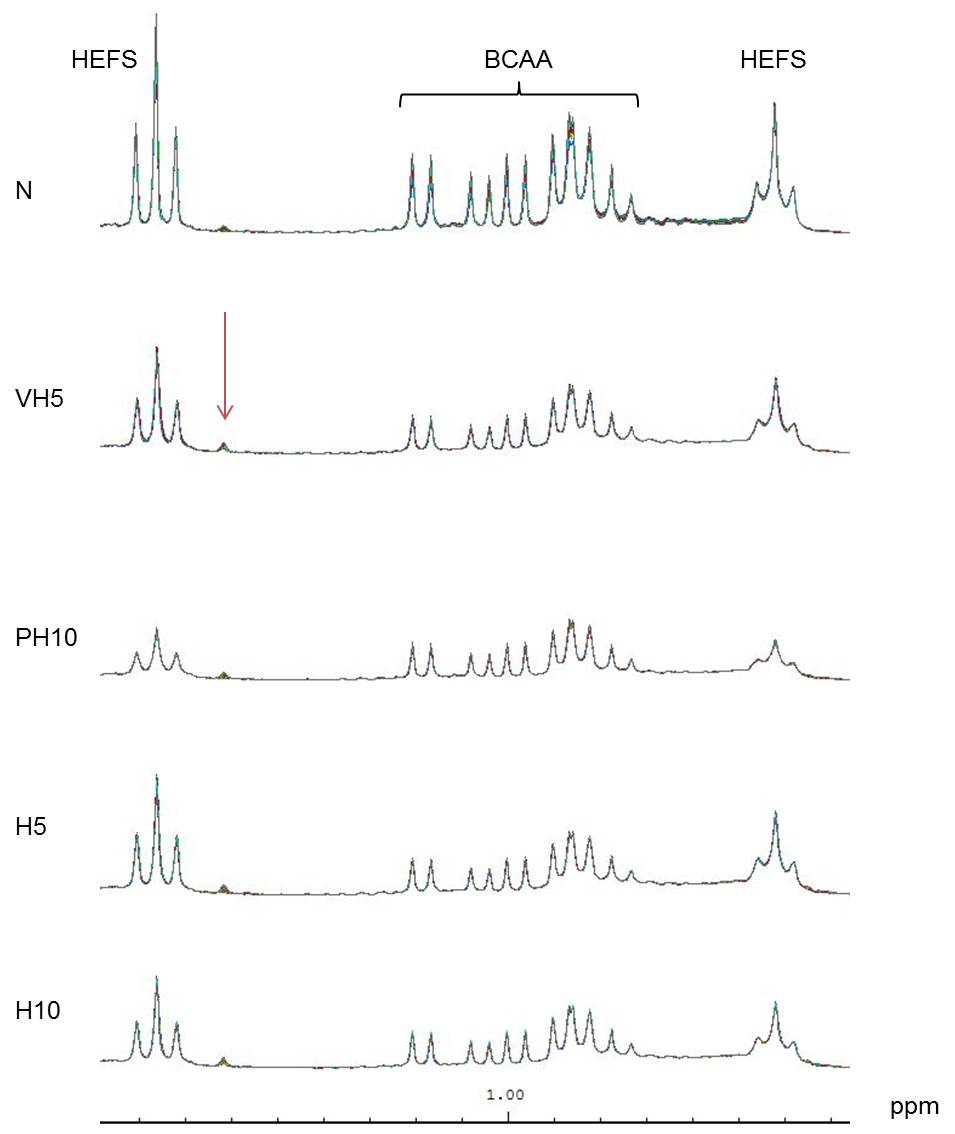


Figure S11. A small portion of the aliphatic region of ^1^H NMR spectra from worm control material extracted by a chloroform/methanol/water (CMW) protocol, treated with heat in order to halt metabolome degradation and repeatedly analyzed over a four day period. The arrow shows the unknown metabolite at 1.15 ppm that has the most dramatic time dependent changes in heated samples. Spectra were normalized to the TMSP peak intensity (0.0 ppm). BCAA: branched chain amino acids.

**References**
